# International Consortium of Rice Mutagenesis: resources and beyond

**DOI:** 10.1186/1939-8433-6-39

**Published:** 2013-12-17

**Authors:** Fu-Jin Wei, Gaëtan Droc, Emmanuel Guiderdoni, Yue-ie C Hsing

**Affiliations:** 1 Institute of Plant and Microbial Biology, Academia Sinica, Hsing: Rm312, IPMB, Academia Sinica, Nankang District, Taipei 11529 Taiwan; 2 CIRAD, Centre de coopération Internationale en Recherche Agronomique pour le Développement, Cirad - av. Agropolis -TA A-108/03, 34398 Montpellier Cedex 5, France

**Keywords:** Functional genomics, Mutants, Rice

## Abstract

Rice is one of the most important crops in the world. The rice community needs to cooperate and share efforts and resources so that we can understand the functions of rice genes, especially those with a role in important agronomical traits, for application in agricultural production. Mutation is a major source of genetic variation that can be used for studying gene function. We will present here the status of mutant collections affected in a random manner by physical/chemical and insertion mutageneses.

As of early September 2013, a total of 447, 919 flanking sequence tags from rice mutant libraries with T-DNA, *Ac/Ds*, *En/Spm*, *Tos17, nDART/aDART* insertions have been collected and publicly available. From these, 336,262 sequences are precisely positioned on the japonica rice chromosomes, and 67.5% are in gene interval. We discuss the genome coverage and preference of the insertion, issues limiting the exchange and use of the current collections, as well as new and improved resources. We propose a call to renew all mutant populations as soon as possible. We also suggest that a common web portal should be established for ordering seeds.

## Introduction

Rice (*Oryza sativa*) is one of the most important crops in the world. Rice, wheat, and maize together account for 60% of the world’s food production, and rice is the principal food of nearly 50% of the world’s population. These cereal crops share a large degree of synteny, so rice is an excellent model cereal crop for genomics research (Gale and Devos 1998). In addition, because of its small genome size, high transformation efficiency and huge genetic resources, rice was the first crop plant chosen for complete genome sequencing.

The world’s population will be greater than 9 billion in less than 40 years. How can farmers grow enough food to feed such a large population in a more sustainable and environmentally friendly way? This “9 billion-people” question is one of the world’s most pressing issues and needs to be solved soon so that we can supply farmers with the seeds to feed future generations. Recently Qifa Zhang and colleagues proposed “Rice 2020” with a goal to assign a biological function to each identified gene by 2020 (Zhang et al. 2008;Zhang and Wing 2013). Rod Wing then proposed the “9-billion project” to solve the 9-billion people problem at the 10^th^ International Symposium of Rice Functional Genomics meeting held in Chiang Mai, Thailand in 2012. To fulfill this important responsibility, the rice community needs to cooperate and share efforts and resources so that we can understand the functions of rice genes, especially those with a role in important agronomical traits, for application in agricultural production.

With the complete genomic sequencing of rice (IRGSP 2005), the challenge of the post-genomic era is to systematically analyze the functions of all genes in the genome. An important and direct approach to defining the function of a novel gene is to abolish or activate its function by mutagenesis. Insertional mutagenesis, with T-DNA or a transposable element, provides opportunities for assigning a function to a particular DNA sequence and isolating the target gene causing a specific phenotype. The function of a gene can be explored efficiently by use of traditional mutagenesis with physical or chemical mutagens and modern tools such as targeting-induced local lesions in genomes (TILLING) and next-generation sequencing (NGS). Many review papers, including three recent ones(Droc et al. 2013;Wang et al. 2013;Yang et al. 2013), have introduced the rice mutant resources and their application. In this current review, we focus on the limitations, characterization and re-analysis of current resources as well as the creation of new improved resources.

### Current status of international mutant collections

Mutation is a major source of genetic variation and may be used for gene functional analysis. Changes in the gene status of a rice plant can be driven in a random manner, by disruption via physical (Bruce et al. 2009;Wu et al. 2005) (such as fast neutron, γ-ray, ion beam), chemical (Till et al. 2007) such as ethyl methansulfonate [EMS], methyl nitrosourea [MNU], sodium azide [SA]) or insertion mutagenesis (T-DNA, *Ac/Ds*, *En/Spm*, *Tos17, nDART/aDART* (Krishnan et al. 2009).

Insertion mutant collections were generated in the late 1990s at the National Institute of Agrobiological Sciences (NIAS, Japan; Miyao et al. 2003) and at the Pohang University of Technology (POSTECH, Korea; Jeong et al. 2002), making use of the endogenous *Tos17* retroelement and T-DNA insertion, respectively. These two laboratories have generated large collections of insertion lines (50,000 and 105,000 lines, respectively) that have been extensively shared among international research groups and thus allowed for deciphering the function of many genes by forward and reverse genetics strategies. Later, T-DNA insertion mutagenesis initiatives were launched in France (Génoplante consortium involving the public institutions CIRAD, INRA, CNRS and IRD, and private companies Biogemma and Bayer Crop Science [Sallaud et al. 2004;Johnson et al. 2005), China (Hua Zhong Agricultural University, Zhejiang University [Wu et al. 2003; the Beijing Biotechnology Research Institute (Wan et al. 2009) and the Shanghai Institute of Plant Physiology and Ecology [(Fu et al. 2009)]) and Taiwan (Institute of Plant and Microbial Biology, Academia Sinica, and the Taiwan Agricultural Research Institute [Hsing et al. 2007), generating a total of 370,000 additional lines. In parallel, *Ds* and *Spm* maize transposable elements have been introduced in rice on a large scale to create lines carrying new transposon inserts in Korea (Plant Molecular Biology & Biotechnology Research Center (PMBBRC); Kim et al. 2004), Australia (CSIRO; Upadhyaya et al. 2006), Europe (EU OSTID consortium; van Enckevort et al. 2005), Singapore (Temasek Life Science; Jiang et al. 2007), and the United States (Cornell University [He et al. 2007;UC Davis [Kumar et al. 2005), with a total of about 150,000 lines. These resources include several functions such as knockout, gene trap, enhancer trap, and/or activation-tag. Most of the flanking sequence tags (FSTs) are searchable at the RiceGE (http://signal.salk.edu/cgi-bin/RiceGE) and OrygenesDB (http://orygenesdb.cirad.fr) websites. As of early September 2013, RiceGE contained 370,179 entries.

The maize *Ac/Ds* transposon system has been used to generate an insertional mutant population in maize itself, Arabidopsis, and rice. An *Ac/Ds*-based library has several advantages: 1) revertants can be readily obtained and easily identified, and 2) because the *Ds* elements prefer to transpose to genetically linked sites (i.e., the same chromosome, Greco et al. 2003;Wan et al. 2009;Kim et al. 2004), an indexed, insertional mutant library can be created with a series of starter lines.

*Ac/Ds* belong to the *hAT* super family, with the designation *hAT* from the *Drosophila melanogaster* element *hobo*, maize element *Ac*, and *Antirrhinum majus Tam3* element. New rice *hAT* elements have been found and are suggested as new candidates to generate insertion mutants in rice. An active 0.6-kb endogenous DNA transposon, *nonautonomous DNA-based active rice transposon1 (nDart1*), was recently identified to act as a causative fragment. For instance, the somatic excision of *nDART1* integrated into a nuclear-coded chloroplast protease led to color sectors in seedlings (Nishimura et al. 2008). Likewise, the integration of *nDart1* into an unknown nuclear protein caused changes in panicle morphology (Yoshida et al. 2013). Thus, this *nDart/aDart* system may be used to generate a large-scale insertional mutant population. Recently, another potential rice *hAT* element, *dTok*, was found by the identification of gene responsible for a mutant with multiple pistils and stamens (Moon et al. 2006).

*Ac*, *Ds* and *Spm* are maize elements. They are introduced by *Agrobacterium*-mediated transformation and these regenerated rice lines may exhibit somaclonal variation especially in the first generation, since the variation will tend to be diluted with no further changes in the offspring, once the mutants are crossed with the *Ac* lines and insert population amplified. Another drawback is the GM nature of the lines. Though belonging to the same *hAT* family as *Ac/Ds*, the use of new *hAT* elements are not prone to these concerns because they are endogenous elements in the rice genome and their mobilization does not necessitate callus formation. Indeed, the the transposition of *nDart1* can be triggered by ordinary crossing under natural field conditions. As for Ac/Ds, the remobilization of the element would generate a revertant and thus may avoid follow-up complementation experiments.

Another important resource to identify useful rice genes is the creation of gain of function lines through the systematic overexpression of full-length cDNAs. This system called gene-hunting (FOX HUNTING) has been used by Kondou and colleagues by a joint effort of RIKEN and NIAS in Japan (Kondou et al. 2009). This group systematically produced 23,000 independent *Arabidopsis* transgenic lines that ectopically expressed rice full-length cDNA. By analyzing the heterologous system, they obtained more than 1,200 morphological mutant candidate lines*.* The same group has also systematically over expressed rice FL cDNAs in rice (Nakamura et al. 2007). Several groups, including researchers in Japan and Korea, used this gain-of-function system to elucidate the novel functions of several rice genes that play important roles in biotic and abiotic stress, seed and root morphology, and pigment accumulation, etc.

The insertion mutants have long been considered as the most user-friendly and prefered functional analysis resource because they contain molecular tags of known sequence and thus integration site information may be readily retrieved. However, with the new technologies such as TILLING and NGS, this advantage has become less conspicuous. Both chemical and ionizing radiation mutagenesis have been routinely used to generate genetic variability in rice varieties since the 1950s; examples are the IRRI effort with IR64 (e.g., Wu et al. 2005) and the Kyushu University effort with Taichung 65, Nipponbare and Kinmaze (e.g., Satoh and Omura 1979). Recently, institutes in Taiwan, Japan, the United States, China, and Brazil have used EMS, MNU, SA, γ-ray, and ion beam to prepare more mutant populations. The genome changes caused by these mutagens include SNP, small indel, large indel, TE transposition, and epigenetic changes. Such differences between wild type and mutant have been discovered efficiently in recent years by using NGS sequencing of bulked DNA of mutant F2 progeny from crosses between mutant lines and parental lines in several plant and animal systems. The first successful case in rice was using MutMap strategy (Abe et al., 2012). The EMS-induced mutant was crossed directly to the original wild-type line, selfed, and the bulked homozygous F2 DNA was subjected to NGS. Because the SNP responsible for the change of phenotype is homozygous, the authors monitored the SNP rates along the chromosomes, and found out followed by confirmation of the target gene. In some cases, the mutant genome sequence is quite different from the RefSeq and the candidate SNP region may be only present in the resequenced cultivar. Takagi and the colleagues then suggested a modified version, MutMap-Gap (Takagi et al., 2013). In such “gap” region, the authors performed assembly and alignment after MutMap method, and identified as well as confirmed the target gene. Recently, this group suggested another modified version – MutMap + where they used the bulked DNA of mutant and wild-type progeny of M3 generation derived from selfing of an M2 heterozygous individual. That is, no cross between mutant and wild-type parental line is required (Fekih et al., 2013).

Table 1 lists the 20 mutant collections available world-wide, associated information, and whether the phenomics database is available. We list several resources not listed in other recent reviews, including the ones at Cornell University, the Taiwan Agriculture Research Institute, the Chinese Academy of Agricultural Science, and the Brazil Plant Genomics and Breeding Center. Most of the 20 collections are japonica varieties, including Nipponbare (used in 11 resources), Tainung 67 (2), Dongjin (2), Zhonghua 11 (2), and Kitaano, Zhonghua 15, Hwayoung, Kitaake, Taichung 65, Yukihikari, Kinmaze, and BRS Querencia (1 each). Some of the collections are indica rice, including IR64 (2), SSBM (1), and Kasalath (1). Of note, indica rice represents about 80% of the world’s rice production in terms of yield or cultivated area. However, more efforts have been invested in producing mutants of japonica rice.

**Table 1:** Current status of rice mutant collections

**Library**	**Institute**	**Genetic background**	**Mutagen**	**Phenomics database**	**Website and/or email**
POSTECH Rice Insertion Database (RISD)	Pohang University of Technology and Kyung Hee University, Korea	Dongjin, Hwayoung Kitaake	T-DNA GT, AT	N/A	http://cbi.khu.ac.kr/ genean@khu.ac.kr
Rice Mutant Database (RMD)	Huazhong Agricultural Univ, Zhejiang University, China	Zhonghua 11 Zhonghua 15 Nipponbare	T-DNA ET *Tos17*	Yes	http://rmd.ncpgr.cn/ cywu@mail.hzau.edu.cn
Taiwan Rice Insertion Mutant (TRIM)	Academia Sinica, Taiwan	Tainung 67	T-DNA AT	Yes	http://trim.sinica.edu.tw bohsing@gate.sinica.ed.tw
Oryza Tag Line (OTL) Génoplante	CIRAD-INRA-IRD-CNRS, France	Nipponbare	T-DNA ET (+*Ds*) *Tos17*	Yes	http://oryzatagline.cirad.fr/ emmanuel.guiderdoni@cirad.fr
Shanghai Insertion Population (ShIP)	SIPPE, China	Zhonghua 11	T-DNA ET	N/A	http://ship.plantsignal.cn/home.do ship@sibs.ac.cn
Chinese Academy of Agricultural Sciences (CAAS)	Chinese Academy of Agricultural Sciences (CAAS), Beijing	Nipponbare	T-DNA AT	N/A	tiegang@caas.net.cn
*Tos17* insertion database	National Institute of Agrobiological Sciences, Japan	Nipponbare	*Tos17*	Yes	http://tos.nias.affrc.go.jp miyao@affrc.go.jp
CSIRO	CCSIRO plant Industry, Australia	Nipponbare	Ac/*Ds*, GT/ET	N/A	http://www.csiro.au/pi narayana.upadhyaya@csiro.au
EU-OSTID	European Consortium	Nipponbare	Ac/Ds ET	N/A	http://orygenesdb.cirad.fr/ emmanuel.guiderdoni@cirad.fr
Temasek Ds	Temasek Life Sciences, Singapore	Nipponbare	Ac/Ds GT	N/A	sri@tll.org.sg
UC Davis (UCD) Ac/Ds and En/Spm populations	UC Davis, USA	Nipponbare	Ac/Ds GT En/Spm Ac/Ds AT	N/A	http://www-plb.ucdavis.edu/Labs/sundar/ sundar@ucdavis.edu
Plant Molecular Biology & Biotechnology Research Center (PMBBRC), Korea	Plant Molecular Biology & Biotechnology Research Center, Korea	Dongjin	Ac/Ds GT	Yes	cdhan@nongae.gsnu.ac.kr
Cornell University	Cornell University, USA	Nipponbare	Ac/*Ds*	N/A	Genomics (2007) 89: 532–540.
IR64 deletion mutant population	International Rice Research Institute, The Philippines	IR64	Fast neutron γ-ray, DEB, EMS	Yes	http://irfgc.irri.org/cgi-bin/gbrowse/IR64_deletion_mutants/ H.Leung@cgiar.org
Oryzabase	National Institute of Genetics, Japan	TC65, Yukihikari Kitaano, Kinmaze	MNU, EMS	Yes	http://www.nig.ac.jp/labs/PlantGen/english/oryzabase-e/ nkurata@lab.nig.ac.jp
Taiwan Agriculture Research Institute (TARI)	Taiwan Agriculture Research Institute, Taiwan	Tainung67, IR64	SA EMS	N/A	http://www.tari.gov.tw/english/ wuypei@dns.caes.gov.tw
National Institute for Agrobiological Sciences (NIAS) mutant population	National Institute for Agrobiological Sciences, Japan	Nipponbare	γ-ray ion beam	N/A	nisimura@affrc.go.jp
UC Davis (UCD) TILLING population	UC Davis, USA	Nipponbare	SA, MNU	N/A	http://tilling.ucdavis.edu/ lcomai@ucdavis.edu
Zhejiang mutant population	Zhejiang University, China	Kasalath SSBM	γ-ray EMS	N/A	http://www.genomics.zju.edu.cn/ricetdna.html clspwu@zju.edu.cn
Plant Genomics and Breeding Center	Plant Genomics and Breeding Center, Brazil	BRS Querencia	EMS	N/A	antonio.oliveira@pq.cnpq.br

Long hairpin RNA (hpRNA) technology has been used recently to induce large-scale gene silencing in rice plants (Wang et al. 2013). The investigators used an improved rolling-circle, amplification-mediated hpRNA method to produce a large number of hpRNA constructs simultaneously from 200- to 400-bp, 400- to 600-bp, and 600- to 1000-bp cDNA libraries. Thousands of transgenic hpRNA lines were produced. More than 50% of these transgenic lines showed visible phenotypes, including poor growth, sterility and alteration in plant morphology, seed size, panicles, and heading time. This proportion is much higher than that reported for T-DNA insertion populations (Chern et al. 2007;Lorieux et al. 2012) or *Tos17* insertion populations (Hirochika et al. 2004). Such high efficiency may be caused by 1) all the hpRNA constructs targeting an exon sequence but T-DNA or *Tos17* integrated randomly in chromosomes and 2) hpRNA possibly causing the silencing of a gene family instead of only a single gene. However, this method still involves a callus growth and transformation process, and thus somaclonal variation is still the concern.

### Genome coverage of sequenced-indexed inserts

International implementation of high-throughput PCR-based methods has allowed for the amplification and sequencing of genomic regions flanking insertion sites of T-DNA, *Tos17*, *Ds* and *dSpm* inserts. In total, 447,919 sequence flanking tags have been released in public databases over the past decade. From these, 336,262 sequence-indexed inserts are precisely positioned on the japonica rice chromosomes (cv. Nipponbare, MSU v7.0 release) (Table 2). Many more PCR products were sequenced notably from T-DNA inserts but proven to be T-DNA or backbone sequences. Examination of the anchored FSTs has provided the community with a better understanding of the insertion behaviour of the different types of mutagens in the rice genome. The insertion preference deduced from these analyses may be slightly biased. For T-DNA inserts, the selectable marker gene has to fall into a genomic region favourable for recovery of gene expression. For class II transposon insertions, the position of remobilized inserts might depend on the position of a generally limited number of initial launching pads in turn determined by T-DNA behaviour. Whatever the mutagen, insertions are not evenly distributed along each chromosome. Insertion frequencies tend to be higher at the distal, sub-telomeric and euchromatic regions, which are generally gene-rich regions and lower in heterochromatic regions (e.g., those close to centromeres). Chromosomes with a larger proportion of heterochromatic DNA tend to show a lower insertion density than larger “euchromatic” chromosomes, which generally contain a high density of predicted genes. At a more local scale, “hot” and “cold” spots for integration are observed, notably for *Tos17*.

**Table 2:** **Distribution of sequence-indexed inserts among the 12 rice chromosomes (Updated from Droc et al.**^2013^**)**

**Chr**	**Length(bp)**	**Predicted genes**	**Predicted gene density/Mb**	**Ds**	**Ds density/Mb**	**dSpm**	**dSpm density/Mb**	**T-DNA**	**T-DNA density/Mb**	**Tos17**	**Tos17 density/Mb**
Os01	43270923	5069	117.15	1057	24.43	1509	34.87	29600	684.06	12260	283.33
Os02	35937250	4134	115.03	951	26.46	2088	58.10	23549	655.28	11609	323.04
Os03	36413819	4386	120.45	977	26.83	1539	42.26	27987	768.58	10267	281.95
Os04	35502694	3416	96.22	871	24.53	1146	32.28	18737	527.76	8226	231.70
Os05	29958434	3116	104.01	343	11.45	751	25.07	15467	516.28	7924	264.50
Os06	31248787	3232	103.43	424	13.57	821	26.27	15297	489.52	8668	277.39
Os07	29697621	3061	103.07	431	14.51	746	25.12	14571	490.65	11711	394.34
Os08	28443022	2759	97.00	517	18.18	670	23.56	13151	462.36	6777	238.27
Os09	23012720	2259	98.16	287	12.47	684	29.72	11878	516.15	6196	269.24
Os10	23207287	2292	98.76	445	19.18	604	26.03	11303	487.05	10288	443.31
Os11	29021106	2704	93.17	473	16.30	723	24.91	17544	604.53	6038	208.06
Os12	27531856	2438	88.55	412	14.96	640	23.25	11492	417.41	6533	237.29
	**373245519**	**38866**	**104.13**	**7188**	**19.26**	**11921**	**31.94**	**210576**	**564.18**	**106497**	**285.33**

T-DNA insertions are found at low frequency in repeated DNA and transposable-element (TE)–related sequences at high frequency in gene-rich regions. Gene intervals contain 48% to 63% of the T-DNA inserts, with a strong bias toward the 5' upstream and 3' downstream regions of genes (Sallaud et al. 2004;Jeong et al. 2006;Zhang et al. 2007). *Tos17* inserts are in gene intervals, preferably introns and exons, at a higher frequency (75-85%) than T-DNA inserts. *Tos17* also exhibits a clear preference for certain genes: the mean number of sequence-indexed allelic insertions in *Tos17* target genes is ~3 and can be up to >200 alleles in the current NIAS and Oryza Tag Line (OTL) collections (Piffanelli et al. 2007). *Ds* preferentially inserts into genic regions (64-75%), whereas sequence-indexed *dSpm* inserts have a more balanced distribution among intergenic and genic regions, which resembles that of T-DNA inserts. Altogether, these studies have concluded that the different types of mutagens are complementary to saturate the rice genome with insertions.

The latest joint Rice Annotation Project (RAP) and MSU release v7.0 revealed that 226,861 of the 336,262 positioned inserts (67.5%) are in a gene interval spanning from -1000 upstream of the ATG to +300 downstream of the STOP codon of the 38,866 predicted rice genes (Table 3). More than three-fourth (77%; 29,672) of the rice genes are interrupted by at least one sequence-indexed insert. Current insertions interrupt an annotated promoter, 5’ UTR, exon, intron and 3’ UTR sequences with 16%, 2.7%, 19.3%, 20.7% and 8.8% frequency, respectively. So far, less than half (17,696) of the rice genes have 3 or more insertions. Functional analysis of a gene requires several allelic insertions leading to a knockout and exhibiting confluent plant phenotypes. This situation indeed avoids tedious complementation steps or the implementation of alternative techniques such as searching for chemically induced lesions in TILLING mutant populations or RNAi-based gene knockdown experiments.

**Table 3:** **Differential recoveries of the insertion mutagens in compartments of the rice genome (Updated from Droc et al.**^2013^**)**

			**Mapped sequence**		**Intergenic**		**Genic**		**Promoter**		**5 UTR**		**Exon**		**Intron**		**3 UTR**	
**Mutagen**	**Source**	**Nb of sequences**	**Nb**	**%**	**Nb**	**%**	**Nb**	**%**	**Nb**	**%**	**Nb**	**%**	**Nb**	**%**	**Nb**	**%**	**Nb**	**%**
Ds	CSIRO	611	**591**	96.7	**160**	27.1	**432**	73.1	**98**	22.7	**19**	4.4	**175**	40.5	**88**	20.4	**52**	12.0
Ds	OSTID	1380	**1373**	99.5	**402**	29.3	**972**	70.8	**212**	21.8	**34**	3.5	**371**	38.2	**210**	21.6	**145**	14.9
Ds	PMBBRC	1072	**1053**	98.2	**263**	25.0	**791**	75.1	**175**	22.1	**29**	3.7	**310**	39.2	**176**	22.3	**101**	12.8
Ds	UCD	1122	**1101**	98.1	**422**	38.3	**680**	61.8	**140**	20.6	**42**	6.2	**248**	36.5	**166**	24.4	**84**	12.4
Ds	UCD-RGT	3719	**3661**	98.4	**1244**	34.0	**2418**	66.0	**686**	28.4	**125**	5.2	**714**	29.5	**568**	23.5	**325**	13.4
dSpm	UCD	12889	**11921**	92.5	**6238**	52.3	**5684**	47.7	**1622**	28.5	**128**	2.3	**1365**	24.0	**1160**	20.4	**1409**	24.8
T-DNA	OTL	26788	**24716**	92.3	**11336**	45.9	**13380**	54.1	**4490**	33.6	**618**	4.6	**2520**	18.8	**3461**	25.9	**2291**	17.1
T-DNA	Postech	107171	**106011**	98.9	**37232**	35.1	**68779**	64.9	**22308**	32.4	**3600**	5.2	**14505**	21.1	**17799**	25.9	**10567**	15.4
T-DNA	RMD	65085	**31276**	48.1	**16258**	52.0	**15018**	48.0	**5002**	33.3	**720**	4.8	**2865**	19.1	**3881**	25.8	**2550**	17.0
T-DNA	SHIP	12614	**9682**	76.8	**3786**	39.1	**5896**	60.9	**2079**	35.3	**286**	4.9	**989**	16.8	**1289**	21.9	**1253**	21.3
T-DNA	TRIM	38840	**38302**	98.6	**14195**	37.1	**24107**	62.9	**8230**	34.1	**1694**	7.0	**5196**	21.6	**5342**	22.2	**3645**	15.1
Tos17	NIAS	77740	**77683**	99.9	**10029**	12.9	**67654**	87.1	**6744**	10.0	**1349**	2.0	**26985**	39.9	**27239**	40.3	**5337**	7.9
Tos17	OTL	14284	**14160**	99.1	**1937**	13.7	**12223**	86.3	**1014**	8.3	**196**	1.6	**5644**	46.2	**4414**	36.1	**955**	7.8
Tos17	RMD	19062	**14732**	77.3	**5905**	40.1	**8827**	59.9	**945**	10.7	**156**	1.8	**3114**	35.3	**3825**	43.3	**787**	8.9
	**TOTAL**	382377	**336262**		**109407**		**226861**	67.47	**53745**	15.98	**8996**	2.68	**65001**	19.33	**69618**	20.70	**29501**	8.77

The use of PCR-based methods for systematic sequencing of chromosomal regions flanking insertion points has facilitated direct *in silico* access to mutant seed stocks via dedicated and user-friendly genome navigators such as Rice GE and OrygenesDB. The functions of a large range of genes involved in cell and developmental processes and in the plant response to biotic and abiotic environments have been unravelled by *in silico* reverse genetics with mutant resources. An expanding number of genes have also been identified and isolated after disruption or activation by *Tos17*, *Ds* or T-DNA inserts in forward genetics screens. These genes underlie important traits involved in hormone synthesis, cell wall synthesis, leaf anatomy, pollen/anther development, spikelet formation and response to biotic and abiotic stresses. In a recent survey, Jiang and associates (Jiang et al. 2012) estimated that the function of more than 600 rice genes has been experimentally validated.

### Issues limiting the exchange and use of current collections of insertion mutants

In rice, 6 different commercial cultivars have been chosen to generate insertion libraries (the temperate japonicas Nipponbare, Dong Jin, Hwa Young, Zhonghua 11, Zhonghua15, and Tainung 67). This choice was based on the availability of a genome sequence or the popularity and economic significance and tissue-culture amenability of the cultivar. The choice led to the unanticipated implications in adaptation of the cultivars to greenhouse growth conditions (notably in labs with no expertise in rice cultivation) and the constraints of handling several genetic backgrounds in functional investigations. For instance, the reference-sequenced Nipponbare cultivar is highly photoperiod-sensitive and does not flower under long-day conditions. It exhibits indeterminate tillering and a bushy phenotype under long-day conditions (>12 hr) which favours pest and pathogen attacks and eventually the yielding of a poor seed set. Contrasting phenotypes are observed depending on the growth period within a year. These findings must be taken into account when comparing mutants and transgenic lines by always setting a complete range of controls and/or using phytotron conditions.

Temperate japonica seeds have rather short germination ability as compared with seeds of cultivars of other genetic groups, and germination decline must be anticipated in most collections generated more than 10 years ago. This situation may become critical because funding that allowed for the generation of biological resources may not be available again when rejuvenating them. Indeed, seeds must be increased under field experimental conditions, which could be complicated by the GM nature of most of the insertion collections.

Another limitation is the difficulty in exchanging seeds of a crop species: rice seed international exchanges have to be covered by import permits and phytosanitary certificates from national quarantine authorities. The GM nature of the seeds may further complicate the procedure. Some countries ask for the complex detection of pathogenic forms of otherwise rather ubiquitous bacterial genera such as *Pseudomonas*. Immersion of seeds in hot water (55 °C-60 °C for 15 mn) before shipment can be a requisite for eradicating seed-borne nematodes. However, once practiced, this treatment leads to the short-term shelf life of the seeds, which then must be readily sown after treatment. Intellectual property issues impose filing and signing Material Transfer Agreements (MTAs) before seed transfer, which might lead to additional delay or complication in seed delivery.

We lack a unique stock centre gathering duplicate seeds of international rice mutant collections comparable to the Nottingham Stock Centre for Arabidopsis. This dedicated centre has considerably facilitated access to seeds and furthered the integration of insertion lines in research programs in the model dicot. At least a common web portal should be established for ordering seeds of rice insertion lines from different collections. Indeed, scientists beginning to investigate rice have difficulty navigating such a complex internet landscape.

Although the current size of the international collection of insertion lines (675,000) appears sufficient, the level of molecular characterization of this global resource, with only 336,000 sequence-indexed inserts anchored on the rice genome, still lags behind that of Arabidopsis (385,000), which has a 3-fold smaller genome. So far ~30,000 non-TE genes over 39,000 predicted gene sequences (i.e., 76.9%) harbour at least one sequence-indexed insert. Also, both FST characterization and seed multiplication are complex, error-prone processes involving multiple steps that can each be a source of contamination or mislabelling. Therefore, the quality of the library must be assessed by performing quality checks and by the benefit of experience from users. Experience has shown that the indexing of an insert to a correct seed bag is not always accurate and ranges from 60% to 80% reconfirmation rate. We stress again the need for ordering and examining several insertions putatively leading to knockout of a given gene. However, only 60% of the rice genes have at least 2 allelic insertions (Table 4). The scientific community should intensify the FST generation effort. Nevertheless, the organization of the T-DNA inserts in some lines and redundancy of *Tos17* may hamper fast progress in this area.

**Table 4:** **Number of rice genes with 1, 2, 3+ sequenced indexed inserts in international rice mutant collections (Updated from Droc et al.**^2013^**)**

**Mutagen**	**Source**	**No. of rice genes with x inserts**			**Total genes interrupted**	**Inserts/gene average**	**Inserts/gene max**
		**1**	**2**	**3 or more**			
Ds	CSIRO	272	37	8	317	1.19	6
Ds	OSTID	820	35	1	856	1.04	3
Ds	PMBBRC	465	60	15	540	1.22	25
Ds	UCD	243	46	43	332	1.62	14
Ds	RGT-UCD	811	127	95	1033	1.58	44
dSpm	UCD	1803	389	294	2486	1.66	74
T-DNA	OTL	4945	1430	558	6933	1.41	29
T-DNA	Postech	8695	5474	7973	22142	2.50	131
T-DNA	RMD	5237	1449	843	7529	1.53	51
T-DNA	SHIP	1283	335	247	1865	1.65	24
T-DNA	TRIM	8537	2713	1187	12437	1.47	88
Tos17	NIAS	2646	891	1974	5511	4.54	199
Tos17	OTL	1455	430	825	2710	3.33	87
Tos17	RMD	1509	385	594	2488	2.44	81
No of rice genes	38866	6676	5300	17696	29672	4.91	339

We hereafter summarize the current status of rice mutant resources.

1. Most efforts have focused on generation of insertion mutants and less on using chemical or physical mutagens.

2. Japonica rice varieties are used in most of the mutant resources.

3. Few initiatives involve breeders in teams or provide phenomics data.

4. Each group uses different phenotype descriptions and codes for mutant traits. A crosstalk between holders of mutant collections should be promoted so that the mutant traits from different groups may be unified or compared.

5. Seeds produced in some groups do not benefit of good storage facilities. We need a call to renew all mutant populations as soon as possible.

6. Requesting the mutant lines involves a complicated process such as MTA and quarantine.

7. We lack a centralized seed stock center for all collections.

8. Some efforts are focused on finding new ideal tagging elements.

9. Most initiatives active in the 2000s included generating new mutant lines, generating flanking sequence data, and performing phenomics analysis. However, less effort has been invested in generating new lines in recent years. Instead, most groups focus on the characterization of specific mutant lines.

### Further characterization of existing collections

Among the extensive collections shown in Table 1, only four institutions worldwide use the activation tagging (AT) method. As an example, the TRIM collection contains 38,840 FSTs. Of the 38,866 non-TE genes, the putative knocked-out gene number is 18,665 and the putative activated gene number is 27,403. So 48.6% of the genes may be knocked out and 70.5% of the genes may be activated. Therefore, the AT method may affect more genes than classical insertion mutagenesis.

Most, if not all, of the FST information from all insertion mutant population is available in rice browsers RiceGE and OygenesDB. Revealing the effect of the integration for the knocked-out function is straightforward. However, interpreting the AT population is difficult because the affected genes vary by integration direction and distance as well as the construction of each group. For instance, a cassette with 8 copies of the 35S enhancer was located near the left border (LB) of the Tag8 vector used in the TRIM population. Thus, the genes within 15 kb of the LB to the genes within 5 kb of the right border (RB) -- a 20-kb region -- may be activated (Hsing et al. 2007). However, in the CAAS mutant population, the two copies of 35S enhancers were arranged next to the RB of pER38. Thus, genes located 7 kb from the RB may be activated (Wan et al. 2009).

Another important issue in the insertion mutant resource is the low tagging efficiency; that is, the relationship between genotype (integrated/affected gene) and phenotype is very low. So the observed phenotypes may or may not be caused by the integration of the vector. The estimated tagging efficiency was 5% to 10% in the *Tos17* and T-DNA tagged population. A review paper published decades ago concluded that the plant cell culture itself generated genetic variability (i.e., somaclonal variation; (Larkin and Scowcroft 1981). Such variation occurred in culture subclones and in regenerated plants (somaclones). The somaclonal variation, resulting from a sum of genetic and epigenetic changes, might occur during the callus induction, growth, *Agrobacterium* co-culture, and regeneration process. The *Tos17* mutant population had to go through callus induction, growth, and regeneration processes. However, the T-DNA and *Ac/Ds* mutant population had to go through *Agrobacterium* co-culture in addition to these processes.

With high-throughput sequencing, several studies investigated the sequence changes in rice *Tos17* mutant lines or Arabidopsis regenerated plants by NGS strategies. Because clonal-regenerated Arabidopsis plants show poorly understood heritable phenotypic variation, Jiang and coworkers (Jiang et al. 2012) estimated the genome-wide sequence variation using five R1 lines by NGS. Both SNPs and small indels (<2 bp) were discovered and were confirmed by capillary sequencing. These mutations were evenly distributed between the 5 chromosomes. The average theoretical mutations per plant were 122. Thus, the mutation rate was 10.5 ± 1.0 × 10^-7^, about 60- to 350-fold higher than the spontaneous mutation rate. Miyao and associates (Miyao et al. 2011) sequenced three *Tos17* mutant lines, *ttm2*, *ttm5* and *ttm11*, that they had generated. Some of the sequence changes were then validated by the dye-terminator method. The SNPs in these regenerants were distributed on all chromosomes, with the transitions:transversion ratio 1.1. The estimated mutation rate was 1.74 × 10^-6^ base substitutions per site per regeneration. In addition, the authors detected small indels. Using the paired-end read data, they searched against the sequences for 43 transposable elements such as *Tos 17*, *Tos19*, and *mPing* but detected no transposition events except for in *Tos17*. So they concluded that other than the integration of the retrotransposon *Tos17*, SNPs and indels were the major causes of somaclonal variation. Using a similar strategy, Panaud and associates (Sabot et al. 2011) studied the transposition events of another rice *Tos17* mutant line, AB156365. From the paired-end data, in addition to the *Tos17* insertions (11 of them), they detected the transpositions of another 11 LTR retrotransposons and 12 MITE integrations compared with the Nipponbare RefSeq. The conflicting results should be explained by the different batches of seeds used to produce these lines. Although the Nipponbare RefSeq is high quality, the rice seeds would accumulate sequence variation during propagation each year. Thus, SNPs, indels, and transpositions may pre-exist in the seed batch used for transformation.

**Figure 1:**
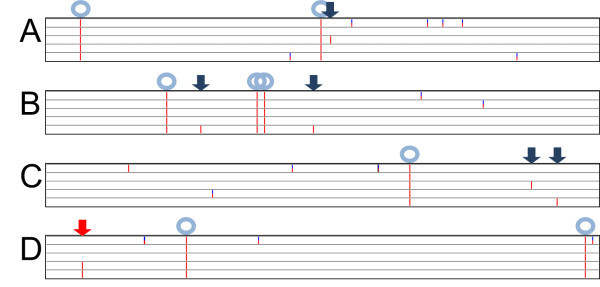
**Single nucleotide polymorphism (SNP) display of TNG67 and 4 TRIM lines. Panel A, B, C, D** show a 100-kb fragment in the rice genome. For each panel, the top lane is the TNG67, followed by M48349, M53677, M79651, M84311, respectively. They are all aligned to Nipponbare RefSeq. SNPs and small indels that are homozygous are indicated as complete red bars and heterozygous as red/blue bars. Dark blue arrows indicate the sequence changes in one of the TRIM lines. Light blue circles indicate the SNP/indel present in TNG67 and thus all 4 TRIM lines. The red arrow indicates the SNP in both M79651 and M84311 but not TNG67 and 2 other mutants.

Figure 1 illustrates the SNPs in the TNG67 and four TRIM lines in four 100-kb fragments. These four mutant lines, M48349, M53677, M79651, M84311, were generated in the transgenic lab at least 4 months apart to ensure that they did not come from the same series of calli. All sequences were aligned to the Nipponbare sequence. Because of the availability of the TNG67 genome sequence, we may differentiate the new SNPs (indicated by blue arrows) and those in the original TNG67 (indicated by light blue circles). The sequencing depth is also important for detecting the real SNPs (complete red bar) or a low-quality read (red-blue bar). Sometimes we see identical SNPs in two different lines (indicated by red arrow) indicating that this variation might already exist in the seeds used for transformation.

Recently Jacobsen and coworkers (Stroud et al. 2013) have shown that rice plants regenerated from tissue culture also harbors stable epigenome changes. Cell dedifferentiation occurring as the first step of cell culture is accompanied with a reprogramming of gene expression which notably relies on a change of DNA methylation status. This group has shown that some of these methylation changes persist in regenerated plants. Notably the observed demethylation of regulatory regions of genes may result in change of expression and thereby of phenotype.

The following are recommendations for solving some of these issues.

1) We should complete the sequencing of all varieties that have been used to generate the mutant populations (i.e., Tainung 67, Dongjin, Zhonghua 11, Kitaano, Zhonghua 15, Hwayoung, and Kitaake). As well, these data should be available in a public domain. They should be used as the reference sequence for detecting changes in the genome rather than Nipponbare.

2) We should pay special attention to spontaneous mutations accumulating in seeds. Although most resource groups received the seeds from rice breeders for transformations, sequence differences could still appear among seeds and among batches. To eliminate this problem, several lines may be sequenced to reveal possible identical SNPs or indels that preexist before transformation. Also it is important to always compare homozygous and azygous siblings in addition to wild type control.

3) Because the activated genes usually provide a dominant trait, we may use the segregation ratio to search for candidate mutant lines caused by integration but not somaclonal variation. The tagging efficiency should be higher. However, this method may be applied only for the Ac population.

4) We may prepare bulk DNA from several lines and perform NGS so that the T-DNA/*Tos17/Ds* integration sites can be discovered efficiently. However, the real SNP or indel and low-quality base readings may not be easy to distinguish for such bulked sample.

### Creating new and improved resources

A call has recently been launched for a shared international effort in the fast generation of an insertion line collection of the temperate japonica cultivar Kitaake (G. An, unpublished). Kitaake is a short-cycling (2 months from seed to seed), temperate japonica variety grown in the northern Hokkaido island of the Japanese archipelago. It exhibits a compact habit, with few but productive tillers and is relatively photoperiod-insensitive (its height varies by day length). Its low tiller number and short cycle allows for high culture density and avoidance of most disease and pest attacks on vegetative and reproductive organs under greenhouse conditions. The Kitaake genome has been recently sequenced (P. Ronald, pers. comm.) and will be soon publicly released; the cultivar is amenable to high-throughput transformation (Kim et al. 2013).

A new, improved gene-trap T-DNA vector should be used, including for example, two T-DNA LBs for preventing backbone transfer as well as, possibly, adjunction of sequences facilitating further flanking-region amplification and sequencing and revealing and avoiding tandem insertions. Also, quantitative PCR screens for selecting single-copy T-DNA events and thereby eliminating multiple, complex and tandem insertions events are now available and can be set up on a large scale to restrict the transfer of useful plants under greenhouse conditions. The success rate in isolating flanking regions is also maximized in the latter events (unpublished), thereby reducing the overall sequencing cost. Also, the efficiency of gene isolation after reporter expression-mediated trapping should be optimized in such a selected sub-population. A common stock center could be set up for this new Kitaake insertion resource, which would facilitate seed orders and expedite regulatory and import permit issues.

Also desirable is avoidance of unwanted DNA lesions and methylation due to somaclonal variation by shorter duration of tissue culture. Fast transformation protocols making use of primary seed embryo-derived calli and no longer their secondary calli have been set up (Toki et al. 2006). Automation of the tricky and tedious washing and decontamination procedure has allowed for increasing the throughput of the method (D. Meynard, unpublished observations). Another objective is the complete avoidance of tissue culture by the development of *in planta* transformation procedures. The *in planta* transformation protocol used in Arabidopsis, floral dipping, was instrumental in establishing insertion libraries, greatly facilitated the integration of routine transformation procedures in laboratory practices, and eliminated variations due to callus culture (although not those created by T-DNA–aborted integrations). In rice, *Agrobacterium-*mediated transformation following the piercing of young seedlings has allowed for the generation of transgenic rice but still not in a routine manner (Supartana et al. 2005;Lin et al. 2009). Establishing a high-throughput *in planta* protocol would be of great interest and would certainly enhance the efficiency of forward genetics screens.

In the past 2 years, novel technologies such as zinc finger nucleases, Transcription Activator-Like Effector (TALE) nucleases or Clustered Regularly Interspaced Short Palindromic Repeats (CRIPSR)/Cas9 have shown promise for generating double-strand breaks and subsequent lesions in genomes of higher organisms (Gaj et al. 2013). TALE nucleases have recently been used for creating mutations in a rice gene involved in the development of bacterial blight disease, with a high frequency of mutation (20–30%), including bi-allelic mutations, at the T0 generation (Li et al. 2012). Recent papers have also shown that rice is amenable to CRISPR Cas9 technology (Shan et al. 2013) making rice the first crop having the genome edited using this method. The ability to generate at a high throughput lesions in the genome guided by sequence-specific single guide (sg) RNA complementary to the target DNA and to multiplex such sgRNA in a single transfection using a universal Cas9 nuclease module opens new avenue to generate novel resources that systematically target genes having no insert or no alleles in existing mutant collections.

Although these methods are experimental and their use is still limited to a few laboratories, the systematic creation of lesions and simultaneous insertion of a reporter in any rice gene may become tractable in the near future in an international effort. Then it might possible to establish an insertion resource that systematically targets rice genes by simultaneously creating both a knockout and a reporter line using these technologies that will greatly facilitates the deciphering of the function of all rice genes by 2020.

## Conclusion

We summarize the present status of rice mutant collections affected in a random manner by physical/chemical and insertion mutageneses. After about 10 years since the first publication on insertional mutant work, the flanking sequence tags of the current world-wide collections reach three quarters of the calculated number for genome saturation and they are publicly available. We point out issues limiting the exchange and use of the current resources, and provide suggestions to solve the problems.

## Abbreviations

EMS: Ethyl methansulfonate; nDart1: *nonautonomous DNA-based active rice transposon*; NGS: Next-generation sequencing; MNU: Methyl nitrosourea; SA: Sodium azide; TE: Transposable element; TILLING: Targeting-induced local lesions in genomes.

## Competing interests

The authors declare that they have no competing interests.

## Authors’ contributions

F.J. W. and G.D. performed the analysis, prepared the tables and figure. E.G. and Y.I.C.H. wrote the paper. All authors read and approve the final manuscript.
